# Nocturnal Acidification: A Coordinating Cue in the *Euprymna scolopes*–*Vibrio fischeri* Symbiosis

**DOI:** 10.3390/ijms23073743

**Published:** 2022-03-29

**Authors:** Brian L. Pipes, Michele K. Nishiguchi

**Affiliations:** Department of Molecular and Cell Biology, University of California, Merced, CA 95343, USA; bpipes@ucmerced.edu

**Keywords:** symbiosis, *Vibrio*, bioluminescence, cephalopod

## Abstract

The *Vibrio fischeri*–*Euprymna scolopes* symbiosis has become a powerful model for the study of specificity, initiation, and maintenance between beneficial bacteria and their eukaryotic partner. In this invertebrate model system, the bacterial symbionts are acquired every generation from the surrounding seawater by newly hatched squid. These symbionts colonize a specialized internal structure called the light organ, which they inhabit for the remainder of the host’s lifetime. The *V. fischeri* population grows and ebbs following a diel cycle, with high cell densities at night producing bioluminescence that helps the host avoid predation during its nocturnal activities. Rhythmic timing of the growth of the symbionts and their production of bioluminescence only at night is critical for maintaining the symbiosis. *V. fischeri* symbionts detect their population densities through a behavior termed quorum-sensing, where they secrete and detect concentrations of autoinducer molecules at high cell density when nocturnal production of bioluminescence begins. In this review, we discuss events that lead up to the nocturnal acidification of the light organ and the cues used for pre-adaptive behaviors that both host and symbiont have evolved. This host–bacterium cross talk is used to coordinate networks of regulatory signals (such as quorum-sensing and bioluminescence) that eventually provide a unique yet stable environment for *V. fischeri* to thrive and be maintained throughout its life history as a successful partner in this dynamic symbiosis.

## 1. Introduction

Eukaryotes continuously encounter a diverse array of bacterial species, some of which they enter with into both short- and long-term symbiotic associations. Though these host–bacteria associations may result in both beneficial and detrimental outcomes, historically research has focused mainly on understanding detrimental pathogenic interactions. Recently, growing evidence indicates that many eukaryotes also establish long-lived, beneficial symbioses with specific bacterial microbiota [1]. These mutualistic symbioses are often essential for initiating and maintaining normal host development and homeostasis [2]. Studies in which hosts were raised without their normal symbiont partners in a bacteria-free environment have revealed that colonization with symbiotic bacteria often is required for normal host development, nutritional metabolism, reproduction, and defense [3,4]. For example, the mammalian gut, where normal colonization by beneficial bacteria provides the host with nutritional benefits, also promotes host immune system development [2], and has become a well-studied model system for research into the cross-talk between beneficial bacteria and their host partners.

Host colonization by beneficial (and pathogenic) bacteria is also a dynamic process, in which bacteria continually sense and respond to the presence of their host [1]. Deeper experimental probing of the cellular and molecular mechanisms beneficial symbionts have evolved to establish and maintain colonization of their hosts has rapidly advanced in the last several decades with the introduction of multiple symbiotic model systems [5]. As more than 97% of all animal species are invertebrates, which maintain some type of beneficial microbial association, there are numerous studied invertebrate–microbe model systems being employed [6,7]. One preeminent example is the Hawaiian bobtail squid (*Euprymna scolopes*) and its bioluminescence bacterial symbiont *Vibrio fischeri* [5,6]. While use of the *V. fischeri–E. scolopes* model system has developed over the past 40 years, invertebrate–microbe model systems have a long history of use in research, dating back to Elie Metchnikoff’s discovery of phagocytosis in starfish larvae in the 1880s [8].

The abundant symbiotic microbial diversity hosted by marine invertebrates plays an important role in their biology and ecology, and resistance to environmental perturbations [7,9,10,11]. Some marine microbial symbionts have been found to have an active role in chemical defenses of their hosts against predators [12]. The marine γ-proteobacterial symbiont *V. fischeri* assists its host squid *E. scolopes*, both in defense from predators and in searching for prey through a fascinating chemical process—the production of bioluminescence [13]. Previous research has led to an increasingly deep understanding of the complex regulation of the rhythmic bioluminescence production observed when *V. fischeri* has colonized a host squid (the celebrated “quorum-sensing” autoinducer pathways were first revealed in this system) [13]. Work with this symbiosis model system has led to greater understanding of the general principles of the evolution and function of more complex symbiotic relationships (such as the symbioses we have with our own microbiome), and has also had pragmatic results, with *V. fischeri* luciferase genes now being a widely used genetic reporter research tool [14]. Here, we will present recent illuminating work with the *V. fischeri*–*E. scolopes* invertebrate model that showcases its utility as a research model system while probing the mechanisms of some of the interconnected regulatory pathways that serve to maintain a stable, mature partnership in the symbiosis.

## 2. Discussion

### 2.1. The Sepiolid Squid–Vibrio fischeri Mutualism

The *V. fischeri*–*E. scolopes* model system has become an important tractable model system for studying associations between animal hosts and bacterial symbionts [15], as it is possible to raise the host and symbiont separately where they can be experimentally manipulated. Within its squid host, *V. fischeri* cells colonize and inhabit a complex organ termed the light organ, where the host squid provides a microenvironment that is environmentally advantageous for the bacteria [16]. In return for shelter and host-secreted nutrients, the bacteria produce bioluminescence, which is used by the squid host for counterillumination during its nocturnal foraging [17]. Counterillumination is a mild ventrally directed countershading light that reduces the squid’s shadow and silhouette produced by nocturnal light (moonlight), allowing the squid to avoid detection by predators and or prey [18,19].

**Figure 1:**
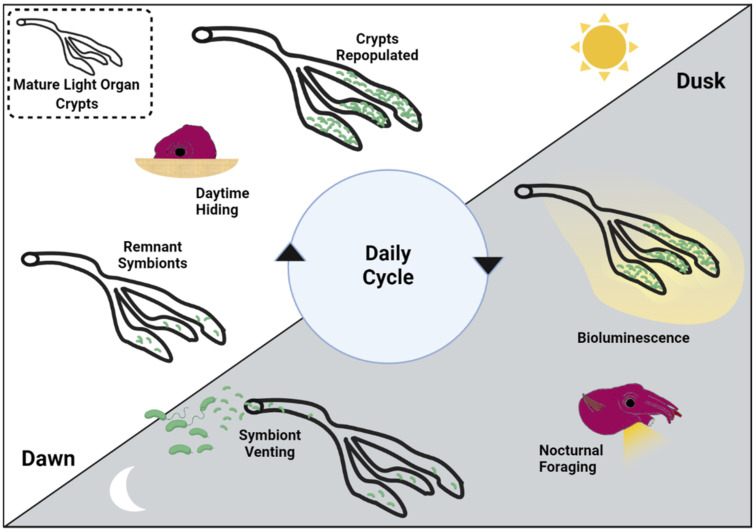
Nocturnal bioluminescence production in the mature light organ. Four weeks after initial symbiont colonization of the light organ a mature diel cycle has developed that will last the life of the squid host. At dawn, 90–95% of the light organ symbionts are vented into the seawater column, where they adopt a planktonic lifestyle until they colonize a newly hatched squid. As the host squid buries itself under the sand and rests during the day, the remaining symbionts rapidly repopulate the light organ. Though high cell densities are reached within a few hours, bioluminescence is not triggered until dusk, when the squid emerges from the sand to forage. At dawn, with bioluminescence no longer needed, most of the symbionts are vented, and the cycle repeats.

The light organs of newly hatched squid are axenic but are colonized within hours by *V. fischeri* from ambient seawater (this is a type of environmental acquisition of symbionts) [20]. These colonized light organs respond to successful colonization with *V. fischeri* by developing an altered mature anatomy, which prevents future light organ colonization for the life of the squid. Bacteria in the colonized light organ replicate rapidly and can reach very high densities (10^9^ cells/adult light organ). The squid host does not maintain this heavy population of symbionts indefinitely; rather expelling 90–95% of the symbionts from the light organ at dawn into the surrounding seawater [21]. The remaining bacteria continue to reproduce in the light organ as the squid host lies buried in sand throughout the day, again reaching the same high cell densities within a few hours. By dusk, the symbionts that have repopulated the light organ begin producing luminescence, which continues until the following dawn. This diel cycle of symbiont growth during the day, bioluminescence production in the light organ at night, and venting of most of the bacteria at dawn continues for the entire life history of the squid [22] (Figure 1), making it the first animal–bacterial symbiosis where such daily rhythms have been identified and extensively studied [23]. Vented *Vibrio* bacteria transition to a free-living lifestyle in the seawater column, where they may at some point recolonize a new juvenile squid host. They can also adapt at this time to a planktonic lifestyle, living in biofilms attached to sediments, suspended particulate matter, and the surfaces of other organisms [22,24].

When symbionts change from a free-living to symbiotic state as they are acquired by each new generation of squid host, they must adapt to often profound changes in ambient conditions, such as pH and nutrient availability [15]. Bacterial symbionts respond to these fluctuating environments through changes in gene expression [16,17,18,19]. At the same time, host tissues adapt their morphology, physiology, metabolism, and immune responses to accommodate the newly acquired symbiont [20,21,22,24,25]. Thus, a stable, shared microenvironment is constructed by both symbiont and host with optimal conditions produced by adaptive signaling between the partners [26,27].

Many of these regulatory signaling networks remain undefined, but research has begun to shed light on the mechanisms of several pathways and has provided tantalizing clues to how both organisms have evolved throughout the history of the mutualism [21,22,27]. One important symbiotic feature in the *V. fischeri*–*E. scolopes* mutualism is the coordinated regulation of luminescence production [28,29].

### 2.2. Bioluminescence in the Light Organ

Bioluminescent bacteria are common members of marine symbioses, whether as members of the enteric microbiota communities, as opportunistic pathogens, or as with *V. fischeri*, as mono-cultures colonizing the light organs of host squids and fish [28]. Often, normal anatomical development of host light organ tissues requires the continuous presence of their species specific luminous bacterial symbionts [29]. In *V. fischeri*, luminescence is produced by the luciferase enzyme, using dissolved molecular oxygen from the surrounding environment to oxidize an aliphatic aldehyde and a reduced flavin mononucleotide, subsequently emitting a photon of blue-green luminescent light [30,31]. In *V. fischeri*, multiple *lux* genes encode the proteins required for luminescence [30]. The *lux* operon, consisting of the genes encoding for the two subunits enzyme luciferase (*luxAB*) genes and accessory proteins required to produce the aldehyde substrate (*luxCDE*), is regulated through the input of multiple sensory pathways, converging on the quorum (high cell density) sensing regulator LuxR [32] and synthesized 3-oxohexanoyl l-homoserine lactone (3-oxo-C6-HSL) autoinducer LuxI [33,34]. Two additional autoinducers, octanoyl l-homoserine lactone (C8-HSL, produced by AinS) and AI-2, modulate the regulation of transcription of the *lux* genes [35,36,37]. *V. fischeri* cells in a colonized light organ continuously secrete these autoinducer molecules, which increase in concentration within the light organ as the population density of the symbiont increases through the early hours of the day. Symbionts monitor the concentrations of these autoinducers as a proxy signal of their cell density—when high cell densities are reached, a cascade of signals initiated by the binding of autoinducers to the symbionts triggers the expression of the *lux* operon and the generation of bioluminescence. For a recent in-depth review of the regulation of the *lux* operon, see [14].

### 2.3. Luminescence Is Required for Maintenance of Successful Colonization

Bioluminescence production is the sine qua non of the symbiotic relationship—the host squid would not benefit from maintaining the symbiosis if the symbiont were to stop producing bioluminescence. Experimental confirmation of the necessity of bioluminescence production to maintain a lasting symbiosis was provided by work that compared the ability of *V. fischeri* mutants (which produced no bioluminescence) defective for either structural (*luxA*) or regulatory (*luxI* or *luxR*) luminescence genes to successfully colonize the light organ of juvenile squid. While these “dark” mutants were able to successfully colonize nascent juvenile light organs, by 48 h post colonization their population levels were severely diminished compared to wild type controls [31]. The reason for the population drops was not determined, although it was not from increased deaths of the colonizing mutants, as vented symbiont numbers during each diel cycle remained consistent between dark mutants and wild type [38]. When juvenile squids were co-inoculated with equal numbers of dark mutants and wild type strains, the dark mutants were outcompeted, with reduced population levels in the light organ compared to wild type controls within 24 h of inoculation. It was observed that the lack of bioluminescence production from dark mutants colonizing the light organ also led to alterations in some, but not all, of the normal developmental changes typically observed in the anatomical and physiological makeup of the epithelial cells lining the surface of the light organ during normal colonization. While no difference was found in the normal progression of apoptotic cell death in the ciliated surface of the light organ early after inoculation, the normal induction of edema of columnar epithelial cells, which results in their transformation into more cuboidal shaped cells, was absent. This anatomical maturation of the lining of the light organ epithelium, which is normally observed within 48 h of inoculation, did not develop in any of the hosts inoculated with any of the three dark mutants. The specific requirement for bioluminescence production to initiate normal host epithelial development was also observed by experiments where juvenile squid inoculated with growth deficient *V. fischeri* strains displayed normal epithelial maturation, but still produced significant levels of bioluminescence [31]. This production of light creates an environment of localized hypoxia in the light organ that induces the normal host epidermal edema [39]. One factor found to mitigate excessive levels of hypoxia in the matrix, which would attenuate luminescence from the O_2_ dependent symbiont luciferase enzymes, is the dramatic acidification of the light organ matrix throughout the nocturnal cycle [39]. Acidification during the evening enhances dissolved O_2_ levels and can subsequently impact several diel rhythmic changes in both host and symbiont physiology.

### 2.4. Light Organ Acidification

Vibrionaceae are found world-wide, in brackish environments with disparate and fluctuating pH conditions reflecting changing organic nutrient load, dissolved organic carbon levels, and active geologic activity [40,41]. While even small pH changes can dramatically impact microbial communities such as the Vibrionaceae, *V. fischeri* has evolved to thrive in the microenvironment of the light organ, where the matrix pH changes dramatically during the diel cycle, gradually decreasing from a pH ~7.0 after venting at dawn, to a pH ~5.5 just prior to the next venting event [42,43]. This is in agreement with experimental work exhibiting several *V. fischeri* strains able to grow in culture between pH 5–10.6 [44]. In a study investigating the impact that global climate change driven acidification of ocean waters (increasing atmospheric CO_2_ leading to increased levels of dissolved CO_2_ that forms carbonic acid) will have upon the *E. scolopes–V. fischeri* symbiosis, it was found that the *V. fischeri* that was experimentally evolved under low pH (6.0 vs. 7.4) growth conditions for multiple generations outcompeted non-evolved ancestor *V. fischeri* strains in colonizing juvenile squid [43]. Additional studies used a similar experimental evolution approach to assess how adaptation to a low pH environment affects *V. fischeri* growth rates and bioluminescence production, and squid colonization demonstrated significant increases in colonization rates, faster generation times, and increased bioluminescence compared to ancestral wild-type strains [45]. These experiments indicate that the ability to handle the stress of growth in low pH environments is an important colonization feature of *V. fischeri* bacteria. However, this raises the question of what causes the diel fluctuation of pH in the light organ and whether this acidification has other roles beyond being a hurdle the symbiont must overcome in order to colonize and be a successful symbiont in the partnership.

Acidification during the evening in mature light organs was found to be triggered by the release of host-derived chitin, a linear polymer of N-acetylglucosamine from localized hemocytes [46]. Concurrently, transcription of *V. fischeri* genes involved in the fermentation of chitin oligosaccharides is increased, leading to the accumulation of acetic acid (due to the bacterial Crabtree effect in the light organ) [15,47]. This is in sharp contrast to the gene expression patterns of the host and symbiont during the day, when the host instead provides alternative nutrient substrates, including amino acids and glycerophospholipids, which the symbionts metabolize via anaerobic respiration with no resulting change in pH [15,48]. This alternating metabolism helps facilitate luminescence during the night, as the lowered pH in the light organ increases the availability of oxygen required for the luciferase-based light production due to the Bohr effect, releasing oxygen from hemocyanin in localized hemocytes [13,49]. Thus, the host’s nocturnal provision of chitin, along with the symbionts’ concurrent metabolic adaptation, rhythmically generates an acidic environmental milieu in the light organ, which in turn serves to regulate bioluminescence production and reinforce the maintenance of the symbiosis.

The diel acidification of the light organ environment may also reinforce the symbiosis by driving the nocturnal increase in *V. fischeri*-derived immunogenic microbe-associated molecular pattern (MAMP) molecules [50]. Squid enzymes, which inactivate these MAMPs, are less active at low pH; thus, diel light organ acidification would increase MAMP levels at night [51,52]. As MAMPs strengthen the establishment of symbiosis by signaling host pattern recognition receptors, it is possible that nocturnal light organ acidification induces rhythmically elevated MAMP signaling to reinforce the maintenance of the symbiosis, analogously to the reinforcement provided by nocturnal bioluminescence production [53].

As a final example of how the nocturnal acidification of the light organ functions in coordinating multiple aspects of both host and symbiont behavior, secreted outer membrane vesicles (OMVs) from *V. fischeri* have been found to be altered by changes in ambient pH [54]. Environmentally transferred beneficial bacteria, such as *V. fischeri,* often alter their behavior and physiology as they move from the surrounding environment to their host and initiate symbiosis [55,56,57]. Similar alterations occur in order to maintain the symbiosis from the initial infection as a naïve juvenile squid to when the host matures over time [58,59,60]. Differences in host environment nutrient content or pH, among other factors, can drive these changes [54,61]. The nocturnal acidification of the light organ, along with changes in host-derived nutrients, such as amino acids, results in coordinated nocturnal alterations in OMV composition and signaling [62]. Increased levels of the outer membrane protein OmpU in the OMVs membrane, along with encapsulated peptidoglycan fragments, microbe-associated molecular patterns (MAMPs) such as lipopolysaccharide, and flagellar proteins, stimulate increased trafficking of hemocytes to the light organ, with the concomitant release of O_2_ and chitin [62]. Changes in ambient pH (from ~8.2 to ~6.0) during the initial colonization of the squid host also triggered analogous changes in the composition of *V. fischeri*-secreted OMVs [54], which also acted as developmental signals to the host to undergo colonization-associated anatomical and physiological changes [63]. OmpU expression in *V. fischeri* was found to be regulated under acidic conditions by the activities of the H-NS and OmpR proteins. Interestingly, H-NS also plays a role in the regulation of many other aspects of *V. fischeri* physiology, including the negative regulation of luminescence through suppression at the LuxR/I *lux* operon promoter sites [64,65], linking light organ acidification, luminescence, and OMV signaling during the nocturnal cycle.

### 2.5. Nocturnal Acidification and the Induction of Competence and Natural Transformation

The *E. scolopes*–*V. fischeri* symbiosis has illuminated several molecular pathways that are dependent upon nocturnal acidification of the mature light organ, and several of these have been linked to the induction of competence and natural transformation. The daily cycle of expulsion of 95% of the population from the light-organ and re-growth of the remaining population exerts a continuous selective pressure on the *V. fischeri* population (which has been suggested to result in increasingly low pH adapted symbionts) [46]. This selective pressure might drive enhanced rates of genetic exchange within the symbiont population, provided these populations are capable of horizontal gene exchange [66]. Horizontal gene exchange through natural competence is a form of bacterial genetic exchange that is often dependent on inducing conditions in the environment [25]. For example, *V. cholerae* and *V. vulnificus* become competent and can be transformed when grown on chitinaceous surfaces, such as shrimp or crab shells [67,68]. Chitin-induced competence is common to many marine vibrio species, perhaps due to their wide-spread ability to metabolize this common nutrient [52]. After chitin was found to be periodically produced in the light organ by the squid host during the diel cycle, a chitin-induced competence in *V. fischeri* was first demonstrated in the same year [69,70]. Observations of competence and natural transformation in the environment has been extensively studied in the congener *V. cholerae* [71,72]. *V. cholerae* uses a type IV competence pilus to acquire environmental DNA and translocate it into the periplasm. DNA is then brought across the inner membrane into the cytoplasm in a single-stranded form [73]. This transforming single-stranded DNA is coated by protective proteins, such as RecA, before integrating into the chromosome via recombination [74]. Induction of competence in *V. cholerae* is centrally coordinated by the transcription factor HapR, which is regulated through the integration of input from multiple quorum and environmental signal-sensing pathways. HapR activity (and hence competence) is low at low cell densities (low quorum) and increases at high cell density (high quorum), or simply exposure to high levels of autoinducers [61,71]. Transcription of *comEA*, which codes for a DNA-binding protein needed for DNA uptake is upregulated by HapR [61,71]. Expression of the secreted nuclease Dns, which degrades extracellular DNA, preventing DNA uptake and competence, is inhibited by HapR [71]. While HapR induces competency after sensing cell density in the environment, the other known key regulator of competency induction, TfoX, senses chitin in the environment. Tfox expression leads to induction of *comEA* and other competence factors [71,75]. Together, HapR and TfoX control further downstream regulators, such the cytidine responsive regulator CytR, that contribute to competence control [73,76,77].

### 2.6. Competence and Natural Transformation in Vibrio fischeri

Similar research in the role of induced competence and natural transformation in the life-cycle of *V. fischeri* has focused on the genetics of competence and its regulation. The known *V. cholerae* competence genes (as explained previously) are largely conserved in other *Vibrio* species, including *V. fischeri* [69,72,78]. Of the 23 genes known or predicted to have roles in *V. cholerae* transformation, 22 were found as homologs in *V. fischeri* [71]. The mere presence of these homologs does not prove that similar competence regulatory pathway functions are present in *V. fischeri*. For example, work on competence in other *Vibrio* species demonstrated that the HapR homolog OpaR is not required for competence of *V. parahaemolyticus*; likewise, the *V. campbellii* homolog LuxR is not always critical for transformation [79]. Elucidation of the regulatory functions of these *V. fischeri* homologs began after experimental induction of competency was found through culturing in the presence of chitin oligosaccharides or by forcing overexpression of the putative competence regulatory factor, TfoX [76]. Recent investigations have found more corresponding regulatory homologs in *V. fischeri*, notably the HapR homolog LitR, several type IV pili structural proteins, and a putative cytidine responsive regulator, CytR [80]. *V. fischeri litR* knockout mutations were found to abrogate competence induction compared to wild type strains [80]. Knockouts of putative *V. fischeri* pilus genes *pilA*, *pilB*, *pilC*, *pilQ*, and *comEA* also abrogated competence induction, suggesting similar roles in transformation [80]. Experiments in which knockouts of the *V. fischeri* putative cytidine-responsive regulator CytR abrogated natural transformation also suggest that environmental cytidine (from degraded DNA) availability is an inducer of competence [80].

Knowledge of several of the major environmental factors that induce in vitro competence in *V. fischeri* and of the signaling pathways involved has led to the development of increasingly powerful research tools for genetic manipulation studies [76,80]. As in *V. cholera* [78], the known cardinal regulators found to induce competence and transformation experimentally in *V. fischeri* (although not consistently) are the presence of environmental chitin or chitin derivatives (or over expression of the chitin-induced tfoX gene), low nutrient availability, the presence of environmental DNA or nucleosides (the repressor of CytR), and high cell density (which activates LitR through quorum-sensing pathways) [80]. Prior sections have highlighted the extensive research demonstrating that two of the cardinal inducers, high cell density (quorum), and environmental chitin, are found within the light organ and fluctuate rhythmically during the diel cycle in a coordinated interacting pattern with luminescence and acidification. Experimental support for the presence of the other two known inducers, low nutrient availability and the presence of environmental DNA or nucleosides, is indirect, but growing. TfoY is a distant *V. fischeri* homolog of TfoX whose role in competence induction remains unclear [81]. Initially it was reported that chitobiose-induced competence depended on TfoY [76]. A conflicting study, however, found no role for TfoY in TfoX-dependent activities in *V. fischeri* or *V. cholerae* [80]. TfoY was, however, found to be involved in the type VI secretion system (T6SS) pathway-mediated killing of competitor bacteria in the environment, which would release extracellular DNA for uptake by competent cells [82]. Recent work [83] found that T6SS-mediated contact-dependent killing used to eliminate competitors during colonization of the *E. scolopes* light organ could only be induced in vitro by providing culturing media of high viscosity and low pH, characteristics found in the mature light organ nocturnal environment (high viscosity resulting in part from exopolysaccharide production upregulated by LitR) [84]. TfoY (and T6SS-mediated killing) induction in the acidic light organ might also function in enhancing DNA availability for natural transformation.

Available environmental DNA and nucleosides would also be released from symbiont deaths in the light organ as a result of nutrient deprivation, which is itself the fourth known inducer of competence and natural transformation. After symbionts have reached high cell density (quorum) during the early hours of the day they are tightly packed in the lumen of the light organ, are non-motile, and not yet luminescent. At dusk, luminescence is triggered, chitin fermentation is driving increasing crypt acidification, and finally, nutrient deprivation at the end of the nocturnal cycle (due to unsustainable population numbers and or reduction of chitin supply) leads to a transient starvation state [85]. Nutrient deprivation during the end stage of the nocturnal cycle has been inferred by transcriptome expression studies, as well as motility and replication recovery studies of vented symbionts [86,87,88].

**Figure 2:**
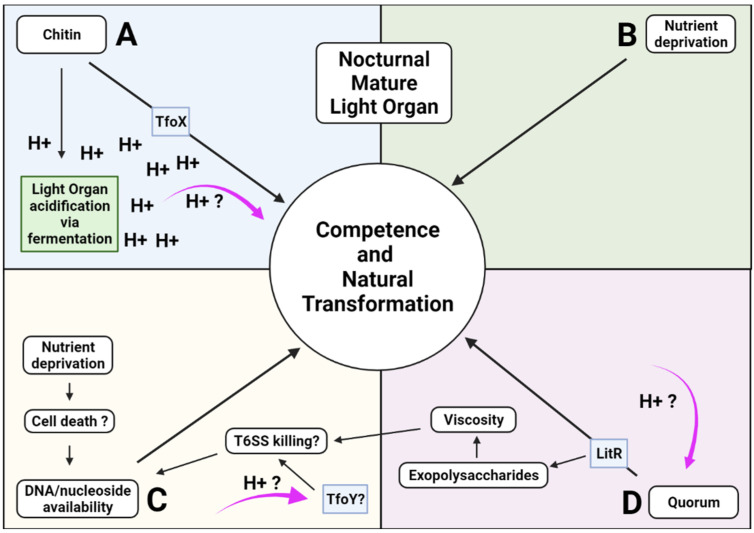
Environmental conditions in the nocturnal light organ conducive to the induction of competence and natural transformation in *V. fischeri* symbionts. The four known cardinal environmental inducers of competence are shown in separate quadrants (**A**–**D**), with arrows indicating induction pathways (not all intermediate pathway enzymes are represented). The putative effects of low pH on the individual induction signals are represented by fuchsia arrows. (**A**) Host-secreted chitin lowers pH via fermentation. Lowered pH may directly act as a competence inducer as it does in some Gram+ species. [89]. (**B**) Nutrient deprivation towards the end of the night has been inferred in the light organ [86,87,88]. (**C**) Nutrient deprivation-induced cell death, along with T6SS-mediated killing, provide a source of DNA/nucleosides. (**D**) Low pH is known to modulate quorum-sensing signaling in some gram positive species [89].

Together, these studies are consistent with the hypothesis that induced competence and natural transformation may be occurring as a result of the specific combination of environmental stimuli (including acidification) present in the light organ during the nocturnal phase of the diel cycle (Figure 2). Further research into the levels of competence and natural transformation induction in *V. fischeri* populations may reveal if nocturnal acidification has additional roles as an inducer or indirect facilitator. Promising work in gram-positive bacterial species has demonstrated that low environmental pH directly induces competence (independent of the four cardinal inducers) and indirectly by regulating the quorum-sensing, demonstrating the ability of environmental bacteria to exchange DNA more readily than was previously thought [89].

### 2.7. Acidification of the Light Organ Acts as a Cue

The nocturnal acidification of the light organ plays a central role in facilitating, coordinating, and maintaining the *E. scolopes–V. fischeri* symbiosis, but this acidification is not a classical signal that the partners respond to directly—it is rather, an indirect environmental cue. Inter-organismal signals evolve to elicit a response in a target organism, with selective pressure for the target organism to evolve a receptor [90]. In contrast, an organism may evolve adaptations in response to a regularly (if not perfectly) recurring chemical or physical cue in its environment to better coordinate its response to subsequent changes in that environment [71]. The nocturnal acidification of the mature light organ may be such a cue that helps maintain the rhythmic timing of host-symbiont behaviors, such as luminescence production, necessary for a successful symbiosis [81]. When the host squid expels the contents of its light organ at dawn, its epithelial lining undergoes morphological and physiological changes that alter the internal milieu (pH rises to neutral, secreted nutrients switch from chitins to glycerophospholipids [46]). The remaining unexpelled symbionts respond to this change in environment by switching to anaerobic respiration of the glycerophospholipids and maximized growth rates [46]. This growth initiates coordinated chemical signaling between host and symbiont that seems to allow preadaptive responses—both host and symbiont adapt their transcriptome profiles before any direct relevant signaling occurs [91]. The high bacterial population level at this time triggers quorum signaling and the start of luminescence production, several hours before it is needed at dusk, while in the host the rising bacterial population has triggered migration of hemocytes into the light organ. Once in the light organ, the hemocytes degrade and release chitin and chitinases, while the epithelial lining cells cease production of amino acid and glycophosphate nutrient supplies. Bacterial chitin fermentation acidifies the light organ, which induces increased O_2_ release from localized hemocytes into the light organ for maximum luciferase (O_2_ dependent) based luminescence production right at dusk when the host squid begins foraging. The quorum-signaling autoinducer AI-2, which is active at this time, may have an unknown role in responding to the acidification cue, as it also regulates responses to environmental stress [92].

**Figure 3:**
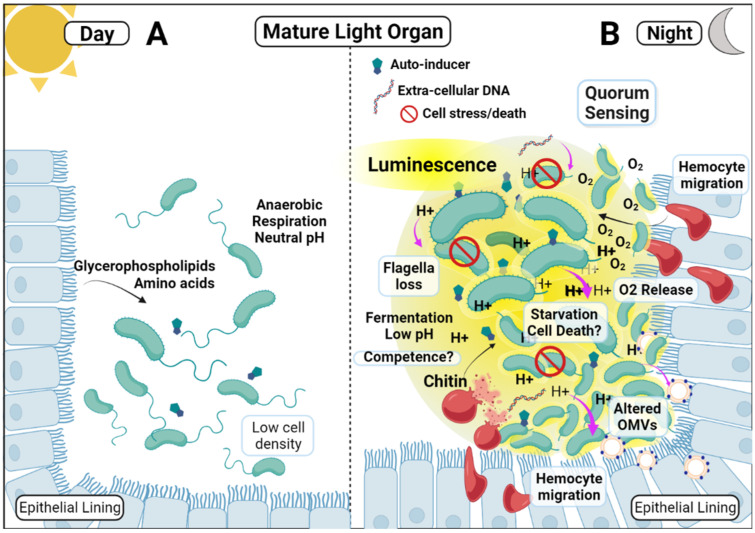
Nocturnal acidification of the mature light organ as a central cue coordinating many of the regulatory signal networks that maintain the symbiosis. (**A**) The matrix of a light organ crypt at dawn when the population of remnant symbionts has not yet repopulated the crypt to a high cell density. Host-provided nutrients are metabolized through anaerobic respiration with no lowering of matrix pH. Flagella may be present, having been induced just prior to the dawn venting. (**B**) Acidification-cued interactions present at the end of the night. Quorum signaling is driving bioluminescence. Fermentation of host-provided chitin lowers the matrix pH and attracts hemocytes which release O_2_. Lowered pH cues production of altered OMVs, while also facilitating several of the four known cardinal inducers of natural competence and transformation. Pre-adaptive flagellar gene transcription is cued shortly before the dawn venting.

The use of light organ acidification as an environmental cue for a rhythmic change in symbiont behavior may also be observed in the loss and regrowth of its flagella throughout the diel cycle. In their planktonic life-stage, *V. fischeri* are motile, with a polar tuft of flagella; yet after successfully colonizing the light organ crypts, flagella are largely absent [93,94]. Such loss of flagellar gene expression has been observed among diverse bacteria that are tightly packed in tissues (similar to the condition of the light organ at night, when *V. fischeri* symbionts are at high cell density) [95]. Flagellar gene expression is known to be repressed by several of the regulators of quorum-sensing and luminescence production (LitR represses flagellin genes, as does AinS [96]), as well as environmental stress response regulators (in *V. cholera*, ArcA represses flagellar gene expression and represses CytR (the negative regulator of competence, which is repressed by free environmental DNA and nucleosides) [97]). Somewhat surprisingly, symbiont transcriptome data has revealed that expression of flagellar structural genes is upregulated several hours before venting at dawn [85,95]. This pre-adaptive response may be rhythmically coordinated via light organ acidification cues coordinating with the timing of the switch of host-provisioned nutrients near dawn. This results in temporary starvation of the symbionts during the latter stages of the nocturnal cycle (nutrient deprivation, triggering the loss of flagella, and conservation of metabolic energy) and has been observed in many γ-proteobacteria species [98]. Acidification cues and the switch to glycerophospholipid nutrients before dawn would then start regrowth of the flagella just prior to expulsion with restored motility shortly (<1 h) thereafter [99]. This type of pre-adaptive response to a changing environment that is likely to be reliably encountered in the future, is a phenomenon known as adaptive prediction [91]. An adaptive prediction response to an encounter with acidic pH cue was also noted during the initiation of the symbiosis, when colonizing *V. fischeri* adhere to the acidic mucus coating the pores leading to the crypts [25]. Coincidentally, an asymmetrical anticipatory predictive switch in *V. fischeri* gene expression occurs, as expression of *eptA* results in positively charged ethanolamine residues being attached to the lipid A component of the cell membrane. This provides protection from antimicrobial peptides, such as polymyxin B, which will only be encountered several hours later inside the light organ crypts [100,101]. In the pre-adaptive anticipatory induction of *eptA* expression at the start of colonization, as in the case of anticipatory flagellar gene expression before venting, the rhythmic acidification of the symbiotic *V. fischeri*’s environment provides a reliable cue for an anticipatory switch for a soon-to-be-needed altered physiological state (Figure 3).

## 3. Conclusions

The sepiolid squid–*Vibrio* partnership is a powerful invertebrate–bacterial model system for better understanding inter-organismal communication in beneficial associations [102]. It is a simple binary open symbiosis where both the host and symbiont can be grown and manipulated separately before introducing them together. The genetic sequence of both the host and symbiont is available, as well as increasingly sophisticated genetic manipulation techniques to answer questions of partner communication and host–symbiont signaling. Investigations into the communication regulating multiple rhythmic diel patterns within the light organ have revealed a complex network of interacting signals and cues which have evolved to maintain a mature stable symbiosis. This review has exemplified the central rhythmic cue of the nocturnal acidification of the light organ, and its interactions in coordinating the myriad of signals which regulate processes such as bioluminescence production, OMV and flagellar production, and possibly natural transformation. Continued work with this model can be expected to reveal the validity of the adaptive prediction concept for many of these rhythmic behaviors. Knowledge from this system and other invertebrate–bacterial symbioses has broad applicability, not only to other beneficial associations, but to the widespread animal epithelium–microbe symbioses with more complex microbial consortia, including the human microbiota [54,103].
